# Dopamine Receptor Antagonists Enhance Proliferation and Neurogenesis of Midbrain Lmx1a-expressing Progenitors

**DOI:** 10.1038/srep26448

**Published:** 2016-06-01

**Authors:** Eva Hedlund, Laure Belnoue, Spyridon Theofilopoulos, Carmen Salto, Chris Bye, Clare Parish, Qiaolin Deng, Banafsheh Kadkhodaei, Johan Ericson, Ernest Arenas, Thomas Perlmann, András Simon

**Affiliations:** 1 Ludwig Institute for Cancer Research, Stockholm Branch, Nobels v 3, 171 77 Stockholm, Sweden; 2 Department of Neuroscience, Karolinska Institutet, Retzius v. 8, 171 77 Stockholm, Sweden; 3 Department of Cell and Molecular Biology, Karolinska Institutet, von Eulers v. 3, 171 77 Stockholm, Sweden; 4 Laboratory of Molecular Neurobiology, Department of Medical Biochemistry and Biophysics, Karolinska Institutet, Retzius v. 8, 171 77 Stockholm, Sweden; 5 Florey Institute for Neuroscience and Mental Health, University of Melbourne, Parkville, Victoria, 3010 Australia

## Abstract

Degeneration of dopamine neurons in the midbrain causes symptoms of the movement disorder, Parkinson disease. Dopamine neurons are generated from proliferating progenitor cells localized in the embryonic ventral midbrain. However, it remains unclear for how long cells with dopamine progenitor character are retained and if there is any potential for reactivation of such cells after cessation of normal dopamine neurogenesis. We show here that cells expressing Lmx1a and other progenitor markers remain in the midbrain aqueductal zone beyond the major dopamine neurogenic period. These cells express dopamine receptors, are located in regions heavily innervated by midbrain dopamine fibres and their proliferation can be stimulated by antagonizing dopamine receptors, ultimately leading to increased neurogenesis *in vivo*. Furthermore, treatment with dopamine receptor antagonists enhances neurogenesis *in vitro*, both from embryonic midbrain progenitors as well as from embryonic stem cells. Altogether our results indicate a potential for reactivation of resident midbrain cells with dopamine progenitor potential beyond the normal period of dopamine neurogenesis.

Midbrain dopamine neurons play key roles in neurotransmission controlling locomotion, cognition and reward. Major motor symptoms in Parkinson’s disease are caused by degeneration of dopamine neurons and an important research aim has therefore been to develop methodology for engineering dopamine neurons from stem cells *in vitro* for cell replacement therapy in Parkinson’s patients^1^,^2^,^3^, reviewed in ref. 4. Detailed understanding of the normal process of dopamine neurogenesis has been essential in these efforts, reviewed in ref. 5. During development, spatially organized signalling events lead to the expression of transcription factors, including the LIM-homeodomain protein Lmx1a in early proliferating neural progenitors localized close to the ventricular wall of the midbrain aqueduct^6^,^7^. Lmx1a, together with the related transcription factor Lmx1b, specify neural progenitors and is essential for the initiation of a molecular program for dopamine neurogenesis^8^,^9^. As Lmx1a-specified progenitor cells exit the cell cycle additional transcription factors are induced, including Nurr1 and Pitx3^5^. These factors promote dopamine neuron differentiation and the acquisition of dopaminergic characteristics as cells migrate first radially and then tangentially towards the prospective ventral tegmental area and substantia nigra^10^,^11^.

The bulk of dopamine neurogenesis occurs normally between embryonic days (E) 10–14 in mice^12^. An exciting possibility would be if substantial dopamine neurogenesis could be induced after the main period of embryonic dopamine neurogenesis. The potential for late dopamine neurogenesis has previously been investigated. Some studies indicate that loss of dopamine neurons in the adult brain evokes responses that could lead to *de novo* generation of dopamine neurons, while others find no evidence for such events^13^,^14^,^15^,^16^. However, both the identity of potential progenitor cells as well as the mechanisms regulating their fate have remained elusive.

Here we addressed the duration of Lmx1a expression in midbrain ventricular cells, and whether Lmx1a-expressing cells could constitute a cell population with progenitor potential for reactivation at later stages of embryogenesis. We also asked if neurogenesis in the developing ventral midbrain could be regulated by the neurotransmitter dopamine itself since previous findings revealed that in aquatic salamanders, dopamine negatively controls the production of dopamine neurons both during homeostasis and regeneration^17^. Salamanders are the only vertebrates known to date with the ability to fully restore the dopaminergic system by a process that is driven by reactivation of dopamine neurogenesis^18^,^19^. Together our analyses indicate a potential for neurogenesis from persisting Lmx1a-expressing cells.

## Results

### Ventral midbrain ventricular cells maintained expression of Lmx1a and other progenitor markers

**Figure 1:**
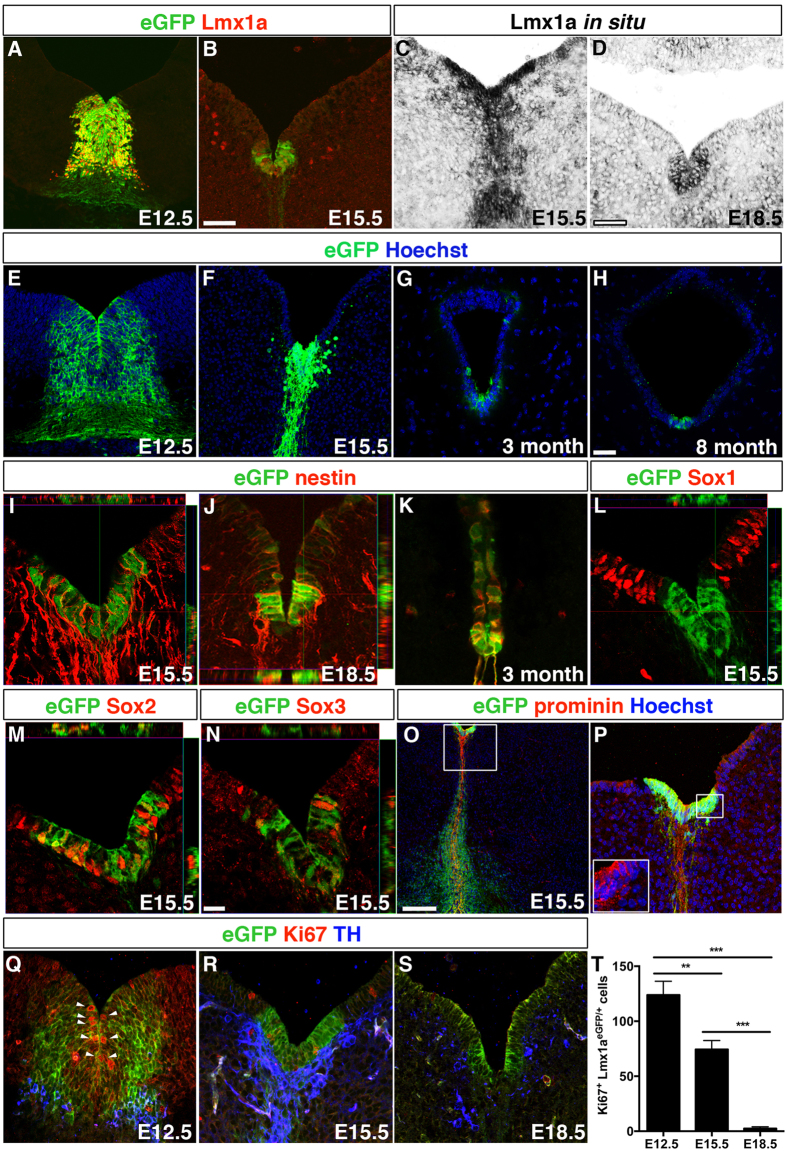
Ventral midbrain Lmx1a-expressing ventricular cells maintained progenitor properties. Lmx1a is expressed in ventral midline cells that generate midbrain dopamine neurons at E12.5, shown by staining against Lmx1a protein and using Lmx1a^GFP/+^ reporter mice (**A**). The expression of Lmx1a was maintained in ventricular progenitors at E15.5 (**B,C**) and E18.5 (**D**) shown by immunofluorescence and *in situ* hybridization. Lmx1a^GFP/+^ reporter mice revealed a persistent presence of eGFP^+^ ventricular cells during development and in the adult animals, depicted at E12.5 (**E**), E15.5 (**F**), 3 months (**G**) and 8 months of age (**H**). However, the eGFP^+^ cells decreased in numbers with time and changed in morphology with a gradual shortening of the processes (**E–H**). Ventral eGFP^+^ ventricular cells expressed nestin both in the embryo and the adult animal (**I–K**). At E15.5 the majority of cells lining the aqueduct were nestin^+^ (**I**), while at E18.5, nestin was restricted mainly to the eGFP^+^ cells (**J**). eGFP^+^ cells were distinguished from other ventricular cells by their lack of Sox1 expression (**L**), but showed overlapping expression with Sox2 (**M**), Sox3 (**N**) and prominin (**O,P**). The number of proliferating eGFP^+^ ventricular cells steadily decreased during development, with 123.9 ± 12.5 Ki67^+^ cells at E12.5 (**Q,T**, n = 6 mice), 74.4 ± 9.1 Ki67^+^ cells at E15.5 (**R,T**, n = 8 mice) and 2.5 ± 1.7 Ki67^+^ cells at E18.5 (**S,T**, n = 5 mice) (**P < 0.01, ***P < 0.001, ANOVA, Tukey’s post hoc test). Scale bars: 50 μM in (**B**) (applicable to **A,K**), 50 μM in (**D**) (applicable to **C**), 50 μM in (**H**) (applicable to **E–G,P,Q–S**), 20 μM in (**N**) (applicable to **I,J,L,M**) and 150 μM in (**O**).

To investigate if Lmx1a expression was temporally restricted to the period of dopamine neurogenesis (E10-E14)^12^ we first performed immunostaining and *in situ* hybridizations at various time points during development. We found that Lmx1a expression was maintained in ventricular cells of the ventral midbrain at E15.5. *In situ* hybridization also demonstrated persistent *Lmx1a* mRNA expression at E15.5 and E18.5 (Fig. 1A–D). However, while Lmx1a protein was expressed at E15.5, no protein expression could be detected by immunohistochemistry at E18.5 and at postnatal stages. We confirmed these findings by examining heterozygous *Lmx1a*^*eGFP*/+^ reporter mouse embryos^8^, which in accordance with immunostaining and *in situ* hybridization showed declining expression, but persistent presence of eGFP^+^ cells throughout development and also in the adult animal at three and eight months of age (Fig. 1E–H).

To characterize ventral eGFP^+^ ventricular cells we analysed the expression of stem and progenitor markers during late embryogenesis. We found that eGFP^+^ cells expressed nestin both in the embryo and the adult animal (Fig. 1I–K). At E15.5 most ventricular cells lining the aqueduct were nestin^+^ (Fig. 1I), while at E18.5, the expression of nestin was restricted mainly to eGFP^+^ cells in the floor plate (FP) (Fig. 1J) and the roof plate (RP) (data not shown). Although nestin was maintained in the adult animal, the morphology of the eGFP^+^ cells changed markedly with age, displaying gradual shortening of their processes, as visualised by eGFP (Fig. 1G,H) and nestin expression (Fig. 1K). In contrast to the neighboring ventricular cells, eGFP^+^ cells were devoid of Sox1 (Fig. 1L), but showed an overlapping expression with Sox2 (Fig. 1M) and Sox3 (Fig. 1N). The Sox1, Sox2 and Sox3 expression patterns were maintained at E18.5 (Supplementary Fig. 1A–F). Notably, the eGFP^+^ cells also expressed the stem cell marker prominin (Fig. 1O,P). Adult ventricular eGFP^+^ cells also displayed a robust expression of Sox2 (Supplementary Fig. 2A–E), indicative of their maintained progenitor potential. In addition, a subset of the adult eGFP^+^ cells expressed Sox3 and prominin (Supplementary Fig. 2F–M). Ki67 staining showed that the number of proliferating eGFP^+^ ventricular cells steadily decreased with developmental age (Fig. 1Q–T) indicating that these cells exited the cell cycle concomitant with the termination of dopamine neurogenesis. Collectively, our data demonstrate that cells of the Lmx1a-lineage maintain progenitor markers during late embryogenesis until E18.5. Thus, dopamine progenitor properties may extend beyond the main period of dopamine neurogenesis.

### eGFP^+^ cells expressed dopamine receptors and were localized within a domain innervated by midbrain dopamine neurons

**Figure 2:**
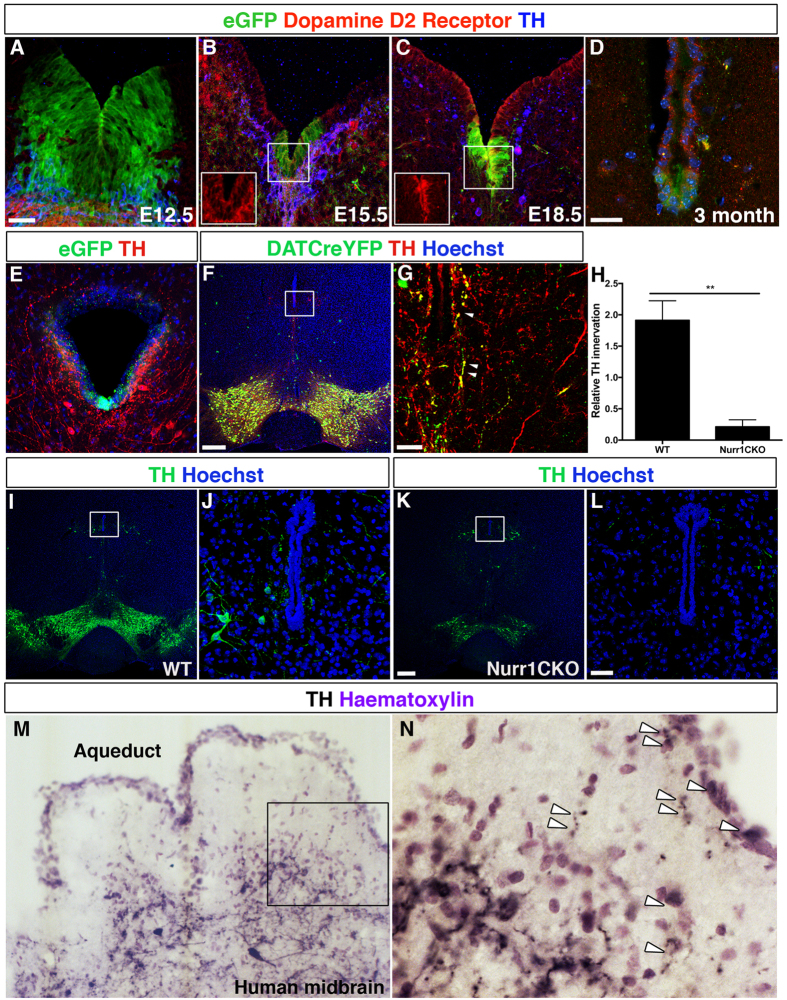
Lmx1a-expressing ventricular cells expressed dopamine receptors and their domain was innervated by midbrain dopamine neurons. There was a gradual increase in the fraction of eGFP^+^ cells expressing D2 receptor during embryonic development (**A–C**). eGFP^+^ cells expressed D2 receptors in adult animals (**D**). Immunofluorescent analysis of 3-month-old mice showed that the ventral aqueduct in the midbrain was innervated by tyrosine hydroxylase (TH^+^) fibers (**E**). Analysis of *DATCre*x*ROSA26YFP* (DATCreYFP) mice demonstrated that TH^+^ fibers surrounding the ventricular eGFP^+^ cells were YFP^+^ and thus of a midbrain dopamine neuron origin (**F,** enlarged in **G**). Periaqueductal TH^+^ neurons, which lack DAT, also extended processes in close proximity to the aqueduct (**F,G**). Nurr1CKO mice, which lack SNc dopamine neurons, and have reduced numbers of VTA dopamine neurons, showed a 7.9-fold reduction in TH^+^ fiber innervation at 5 months of age (*P* = 0.0021, n = 3 + 4, unpaired Student’s t test) (**H–L**). Human midbrain sections demonstrated that TH^+^ fibers were in contact with the outer layers of the aqueductal cells (**M,N**). Scale bars: 50 μM in (**A**) (applicable to **B**,**C**), 50 μM in (**D**), 200 μM in (**F**), 50 μM in (**G**), 200 μM in (**K**) (applicable to **I**), 50 μM in (**L**) (applicable to **E**,**J**).

We previously showed that mature dopamine neurons are in close contact with neural stem cells in the adult salamander, a highly regenerative species in which the dopaminergic system can be completely restored following chemical ablation of dopamine neurons, and in which dopamine negatively regulates midbrain dopamine neurogenesis^17^. We set out to address to what extent such a regulatory mechanism might be evolutionary conserved. First, we found that eGFP^+^ cells expressed dopamine D2 receptors (D2R) both during embryogenesis and in the adult brain (Fig. 2A–D; orthogonal view of E18.5 shown in Supplementary Fig. 3A,B). Next, immunofluorescent analyses revealed that tyrosine hydroxylase (TH^+^) fibers projected onto the ventral aqueduct in the midbrain (Fig. 2E). High magnification confocal images and orthogonal sections indicated that TH fibers partially surrounded a proportion of the eGFP^+^ progenitor cells (Supplementary Fig. 3C,D). To investigate the origin of the TH^+^ fibers projecting to the midbrain aqueduct, we analysed *DATCreYFP* mice, in which YFP is exclusively expressed in dopamine transporter (DAT)-expressing midbrain dopamine neurons. We found that TH^+^ fibers surrounding the ventricular Lmx1a-expressing cells expressed YFP in these embryos, indicating their midbrain origin (Fig. 2F,G). Periaqueductal TH^+^ neurons, located just lateral to the ventral aqueduct, do not express DAT and for this reason did not express YFP in transgenic embryos. These periaqueductal TH^+^ neurons also extended processes in close proximity to the aqueduct (Fig. 2F,G). Histological analysis of conditional Nurr1^DATCre^ (Nurr1CKO) mice, in which most dopamine neurons are lost^20^, showed a 7.9-fold reduction in TH^+^ fiber innervation (Fig. 2H–L) suggesting that a majority of fibers originated from midbrain dopamine neurons in the ventral midbrain area. As periaqueductal TH^+^ neurons are not lost in Nurr1-ablated mice (Fig. 2H,K,L), it is possible that some of the remaining fibers originated from this neuronal subpopulation. Furthermore, we found that eGFP^+^ cells lining the fourth ventricle in the hindbrain were also in close proximity to TH^+^ fibers originating from the locus coeruleus (Supplementary Fig. 3E,F). Notably, analysis showed that TH^+^ fibers were in close proximity with the outer layers of the midbrain aqueductal cells also in the human brain (Fig. 2M,N). Together these data indicated a possibility for dopamine regulation of Lmx1a-expressing ventricular cells.

### Haloperidol increased eGFP^+^ cell proliferation and neurogenesis

**Figure 3:**
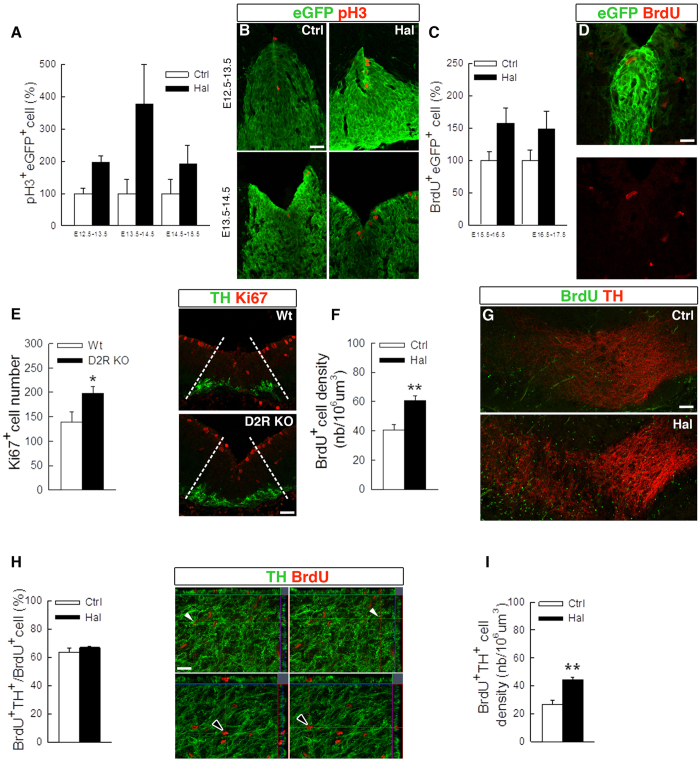
Haloperidol treatment increases the proliferation of eGFP^+^ cells and neurogenesis *in vivo*. Haloperidol treatment increased the percentage of pH3^+^ eGFP^+^ ventricular cells (**A,B**, Haloperidol during E12.5–13.5, n = 8 (Ctrl), n = 9 (Hal); Haloperidol during E13.5–14.5, n = 11 (Ctrl), n = 9 (Hal); Haloperidol during E14.5–15.5, n = 13 (Ctrl), n = 10 (Hal), 2-way-ANOVA, treatment effect *P* = 0,003). Haloperidol treatment increased the percentage of BrdU^+^ eGFP^+^ cells at E16.5 in the ventral part of the third ventricle (**C,D**, Haloperidol during E15.5–16.5, n = 10 (Ctrl), n = 11 (Hal); Haloperidol during E16.5–17.5, n = 12 (Ctrl), n = 12 (Hal), 2-way-ANOVA, treatment effect *P* = 0,016). The number of Ki67^+^ ventricular cells in the ventral midbrain was increased in D2R KO mice at E11.5 compared to wild-type littermates (**E,** n = 5 (Wt), n = 5 (KO), *P* = 0.04, unpaired Student’s t test). Haloperidol treatment at E15.5–E17.5 increased the number of BrdU^+^ cells in the prospective substantia nigra and ventral tegmental area (**F,G**, n = 6 (Ctrl), n = 7 (Hal), *P* = 0.0014, unpaired Student’s t test). Haloperidol treatment at E15.5–E17.5 did not change the percentage of BrdU^+^ cells expressing TH in the prospective substantia nigra and ventral tegmental area (**H**, n = 4 (Ctrl), n = 4 (Hal), *P* = 0.32, unpaired Student’s t test). Orthogonal sections showing BrdU^+^ TH^+^ (top panel, arrow) and BrdU^+^ TH^**−**^ (bottom panel, arrow) **(I)**. Haloperidol treatment at E15.5–E17.5 increased the density of BrdU^+^ TH^+^ cells in the prospective substantia nigra and ventral tegmental area (*P* = 0.002, unpaired Student’s t test). Scale bars: 50 μM in (**B**,**D**,**E**), 100 μM in (**G**) and 20 μM in (**H**).

To examine if eGFP^+^ cells may be regulated by dopamine, we administered the broad-spectrum dopamine receptor antagonist, haloperidol, to pregnant *Lmx1a*^*eGFP*/+^ females. Animals received two daily injections of haloperidol or vehicle and were sacrificed the following day. We first carried out this series of experiments during E12.5–E14.5 and found that haloperidol administration led to an increased number of eGFP^+^ cells that were also positive for the mitotic marker pH3 (Fig. 3A,B). Interestingly, haloperidol induced a particularly strong increase in the number of pH3^+^ eGFP^+^ cells in the caudal portions of the midbrain (Supplementary Fig. 4A). This is in concordance with the more prominent TH innervation in caudal midbrain (Supplementary Fig. 5). We also administered the nucleotide analogue BrdU to animals via osmotic pumps, but positive cells could not be counted because virtually all cells had incorporated the analogue due to the intense proliferation during this active neurogenic period (data not shown). In contrast, when haloperidol was administered after the decline of normal dopamine neurogenesis, we could evaluate BrdU-incorporation and found a 1.5-fold increase of BrdU^+^ eGFP^+^ cells both at E16.5 and E17.5 (Fig. 3C,D). Similarly to the pH3-index, we noted a marked shift towards the caudal portions of the midbrain harbouring more BrdU^+^ eGFP^+^ cells (Supplementary Fig. 4B). We also found that haloperidol appeared to increase the proliferation of the eGFP-negative cells (Supplementary Fig. 4C). In agreement with these data we noted a marked increase in the number of cycling cells in the midbrain progenitor zone at E11.5 in gene targeted mice lacking both alleles of the *D2R* gene (Fig. 3E).

Next we tested whether haloperidol treatment could increase the proliferation of the eGFP^+^ cells and the production of new dopamine neurons after E14.5. We thus administered haloperidol and BrdU between E15.5 to E17.5, as described above, but this time evaluated the number BrdU^+^ cells in the prospective substantia nigra and ventral tegmental area instead of the ventricular zone. A 1.5-fold increase in the density of BrdU^+^ cells was detected in haloperidol treated animals (Fig. 3F,G). These BrdU^+^ cells were equally distributed between VTA and SNc (Supplementary Fig. 4D). Next we analysed the identity of BrdU^+^ cells and found that the percentage of BrdU^+^ cells expressing TH was unaffected, indicating that dopamine neuron differentiation was similar in both groups (Fig. 3H). Hence, the total number of newborn TH^+^ neurons showed a 1.5-fold increase in haloperidol compared to vehicle treated animals (Fig. 3I). These data collectively show that antagonizing dopamine signalling increases the proliferation of ventral aqueductal cells and ultimately the number of newborn TH^+^ neurons in the midbrain.

**Figure 4:**
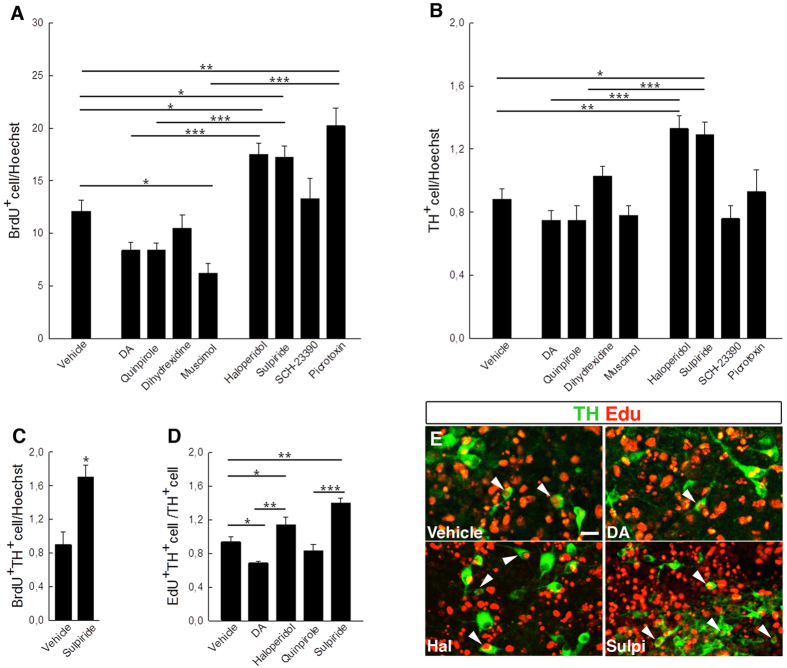
Dopamine receptor antagonists increased cell proliferation and neurogenesis in embryonic midbrain and mESC cultures. The fraction of primary midbrain cells increased significantly upon treatment with the dopamine receptor (DR) antagonists haloperidol and sulpiride, but not with SCH-23390. Dopamine and the DR agonist quinpirole did not increase cell proliferation. The GABA_A_ receptor agonist muscimol decreased proliferation, while the GABA_A_ receptor blocker, picrotoxin, increased proliferation (**A**). Only the DR antagonists haloperidol and sulpiride increased the fraction of TH^+^ cells in primary midbrain cultures (**B**). The DR antagonist sulpiride increased the fraction of BrdU^+^ TH^+^ cells in primary midbrain cultures (**C**). Dopamine (DA) decreased, while haloperidol (Hal) and sulpiride (Sulpi) increased the fraction of EdU^+^ TH^+^/TH^+^ cells derived from mESCs (**D,E**). (**A**,**B**,**D**, *P < 0.05; **P < 0.01; ***P < 0.001, 1-way ANOVA and Newman-Keuls post hoc test, (**C**), unpaired Student’s t test). Scale bars: 20 μM.

To corroborate these findings we tested the effect of various neurotransmitters on primary cultures of embryonic midbrain cells from animals undergoing dopamine neurogenesis. We took advantage of the fact that these cultures contained a mixture of precursor, including both dopamine and γ-aminobutyric acid (GABA) progenitors and used an array of neurotransmitter agonists and antagonists targeting dopamine and GABA receptors. In accordance with the *in vivo* observations we found that haloperidol treatment increased the proliferation of primary embryonic midbrain cells 1.5 fold as assayed by BrdU-incorporation (Fig. 4A). Moreover treatment with the dopamine 2 receptor (D2R) antagonist, sulpiride, but not the dopamine 1 receptor (D1R) antagonist SCH-23390, increased proliferation (Fig. 4A). In contrast, neither dopamine itself nor the dopamine receptor agonists quinpirole or dihydrexidine increased proliferation. We also found that the GABA_A_ receptor agonist muscimol decreased proliferation, while the GABA_A_ receptor blocker, picrotoxin, increased proliferation (Fig. 4A, Supplementary Fig. 6). Consistently with these results we observed an increased fraction of TH^+^ cells in the cultures upon haloperidol and sulpiride treatment, while neither the dopamine receptor agonists nor SCH-23390 had this effect (Fig. 4B). Furthermore, sulpiride also increased the fraction of BrdU^+^ cells expressing TH (Fig. 4C). Although we found that the GABA_A_ receptor blocker, picrotoxin, increased proliferation, the fraction of TH^+^ cells did not increase in the cultures (Fig. 4B), indicating a selective role for dopamine receptor signalling in dopamine neurogenesis. To further validate these observations and to analyse if dopamine signalling could regulate the number of dopamine neurons generated from mouse embryonic stem cells (mESCs) we induced neurogenesis in mESC-derived midbrain cultures and found that dopamine decreased the fraction of EdU^+^/TH^+^ cells whereas both haloperidol and sulpiride increased it (Fig. 4D,E). These *in vitro* data are consistent with the results obtained *in vivo* and indicate that dopamine receptor antagonists may serve as a tool to enhance dopamine neurogenesis.

## Discussion

In this work we characterized ventral midbrain ventricular cells in the Lmx1a lineage through embryogenesis into adulthood in mice. These cells retain expression of nestin, Sox2, Sox3 and prominin beyond the period of normal dopamine neurogenesis during embryonic development and even in the adult, suggesting that they might have sustained progenitor cell properties. We further showed that proliferation and subsequent neurogenic conversion of these cells was enhanced by dopamine receptor antagonist treatment, an effect that extended beyond the bulk of normal embryonic dopamine neurogenesis.

Several previous studies have shown that neurotransmitters influence neurogenesis both during embryonic development and in the adult brain and spinal cord in several species, e.g. in mammals, salamanders and zebrafish^21^,^22^,^23^,^24^. The role of dopamine signalling in neurogenesis in the mammalian brain was addressed in different experimental settings, which collectively indicated region- and cell-type specific effects. Dopamine depletion has been seen to decrease cell proliferation in the dentate gyrus and subventricular zone in some of these studies^25^,^26^,^27^,^28^,^29^,^30^, while others documented increased cell proliferation^13^,^31^,^32^. Furthermore, observations in gerbils and in rats showed increased cell proliferation in dentate gyrus^33^, thus revealing that haloperidol treatment can increase the number of stem cells and the proliferation of transient-amplifying progenitors in the SVZ^34^.

Importantly, the present work is the first to address how modulating dopamine signalling influences cell proliferation and neurogenesis in the developing mammalian midbrain after the peak of dopamine neurogenesis. Although the effects in mice, as shown here, were rather modest, the results are in line with previous findings in aquatic salamanders, which demonstrated increased midbrain dopamine neurogenesis upon haloperidol treatment^17^. This indicates an evolutionary conserved regulatory mechanism by which dopamine suppresses the proliferation of dopamine progenitors and ultimately neurogenesis. Whether dopamine acts in a negative feedback-like manner in the mammalian midbrain remains to be proven. However, it is noteworthy that several reports identify regionally committed stem and progenitor cells that respond to varying levels of neurotransmitters. For example, the neurotransmitter (GABA) negatively controls proliferation of cells in the subventricular zone and in the rostral migratory stream^35^,^36^,^37^, and quiescent neural stem cells respond tonically to GABA by means of γ2-subunit-containing GABA_A_ receptors in the adult dentate gyrus^38^. Hence, a plausible hypothesis is that a function of dopaminergic innervation of Lmx1a-expressing cells could be to maintain progenitors in quiescence, a state that can be transitioned into proliferation upon dopamine receptor antagonist treatment.

Dopamine neurogenesis in the adult salamander midbrain is critically dependent on reduced dopamine levels. Our observations raise the possibility that neurogenesis could be activated from resident Lmx1a-expressing cells by modulation of dopamine receptor signalling also in the postnatal mammalian brain. However, several significant ontogenetic changes occur as the region harbouring these cells transition beyond embryonic neurogenesis. The number of Lmx1a-expressing cells decreases, the expression level of Lmx1a in these cells is decreased and their processes become smaller and do not extend far beyond the aqueduct in the adult brain. As these processes likely support cell migration during development their disappearance may contribute to the diminution of neurogenesis in the adult midbrain. Nevertheless our findings warrant further experiments targeting the neurogenic potential of Lmx1a-expressing population in the adult midbrain.

## Methods

### Ethics statement

All the work involving animals or human subjects/tissues was carried out in accordance with the Code of Ethics of the World Medical Association (Declaration of Helsinki) and with national legislation and institutional guidelines. Animal procedures were approved by the regional animal ethics review board (Stockholms Norra Djurförsöksetiska nämnd) and the Florey Institute of Neuroscience and Mental Health animal ethics committee. Ethical approval for the use of human post mortem samples was obtained from the regional ethical review board in Stockholm, Sweden (Regionala Etikprövningsnämnden, Stockholm, EPN), ethical approval number 2012/111-31/1. All post mortem human tissues were obtained from the National Disease Research Interchange (NDRI, www.ndriresource.org) with the written informed consent from the donors or the next of kin.

### Animals

All mouse lines were maintained on a C57Bl/6 background. The following transgenic mouse lines were utilized: Lmx1a^eGFP/+^ mice^8^; floxNurr1DATCre (Nurr1CKO) mice, generated by crossing floxed Nurr1 mice^20^ with mice carrying Cre inserted into the DAT gene locus^39^; and DATYFPxROSA26 mice, which were generated by crossing B6.129X1-Gt(ROSA)26Sor^tm1(EYFP)cos^/J mice (Jackson laboratory #006148) with DAT-CreERT2 mice; D2R knock-out (D2R KO) mice and wild-type littermates^40^ (Jackson laboratory #003190).

### BrdU delivery through subcutaneous pumps

The subcutaneous pump (Alzet, Model 2001) was filled with BrdU solution (0,08 mg/mL, Sigma), diluted in 60% DMSO and 40% dH_2_O.

### Haloperidol *in vivo* administration

Haloperidol (Sigma) solution was prepared in acetic acid and saline and neutralized by addition of NaOH. For the proliferation experiments, pregnant dams received two daily injections of haloperidol (3 mg/kg) or vehicle at E12.5, E13.5, E14.5, E15.5 or E16.5, and were sacrificed the day after. For chase experiment performed from E15.5 to E18.5, animals were daily injected with vehicle or haloperidol (1.5 mg/kg).

### Mouse tissue processing

Adult mice were anesthetized with avertin (tribromoethanol, Sigma) at 0.5–0.6 mg/g body weight i.p. and perfused intracardially with phosphate buffered saline (PBS) (Invitrogen) and subsequently 4% paraformaldehyde (Sigma). Brains were dissected, postfixed for 2–6 h in 4% PFA, cryoprotected in 20% and then 30% sucrose, sectioned (30–40 μm), serially collected and stored in antifreeze solution (30% glycerol, 30% ethoxyethanol, 40% PBS) at −20 °C until used. Pregnant dams were anesthetised by CO_2_ and sacrificed by cervical dislocation and embryos collected. For immunohistochemistry and *in situ* hybridization, collected embryos were postfixed in 4% PFA (1–3 h depending on the age of the embryo), cryoprotected in 20% and subsequently in 30% sucrose, embedded in OCT (TissueTek), sectioned at 12–20 μm, serially collected and stored at −80 °C.

### Human tissue processing

The tissues were fixed in 4% PFA, sequentially placed through sucrose gradients (2%, 10%, 20% and 30%) for cryoprotection and sectioned on a freezing microtome (Microme HM430) at 40 μm thickness and serially collected. Tissues were subjected to antigen retrieval (0.01 M citric acid buffer, pH 6.0 for 20 min at 95 °C) and blocking of endogenous peroxidases (3% H_2_O_2_ in 50% methanol in PBS) prior to staining^41^.

### Histological procedures for mouse and human tissues

For immunofluorescent staining of mouse tissues, sections were rinsed with PBS and incubated with blocking buffer (PBS, 5–10% normal donkey serum; NDS or normal goat serum; NGS (Jackson Laboratories), 0.1% Triton-X100) for 1 h prior to incubation with primary antibodies (Supplementary Table 1) in blocking buffer overnight at 4 °C. The tissue sections were subsequently incubated in Alexafluor secondary antibodies (1:500) for 1 h, and rinsed in PBS. Hoechst 33342 (4 μg/ml) was used for counterstaining and tissue sections were mounted onto slides in Mowiol 4–88 (Calbiochem). Confocal analysis was performed using a Zeiss LSM510/Meta Station (Thornwood, NY, http://www.zeiss.com). For identification of signal co-localization within a cell, optical thickness was kept to a minimum, and orthogonal reconstructions were obtained. Each experiment was performed in triplicate and all counts were done in a double-blind fashion.

Sections from human postmortem tissues were incubated with blocking buffer for 1 h. Subsequently, sections were incubated overnight at 4 °C using goat anti-tyrosine hydroxylase antibody (1:300, PelFreez). Sections were washed in PBS and incubated with a biotinylated secondary antibody (1:200; Vector Laboratories) for 1 h at room temperature, followed by incubation in streptavidin-biotin complex (Vectastain ABC kit Elite, Vector laboratories) for 1 h and visualized by incubation in 3,3′-diaminobenzidine solution (DAB, Vector Laboratories). Nuclei were counterstained using Myers hematoxylin (Histolab) for 1–2 min, rinsed in ddH_2_0, quickly immersed in 70% ethanol containing 36 mM HCl to remove background staining, followed by another rinse in ddH_2_0. Sections were subsequently dehydrated by sequential steps in increasing ethanol concentration accordingly; 25% ethanol (2 min), 70% ethanol (2 min), differentiator (75% ethanol containing 96 mM acetic acid) (1 min), 95% ethanol (2 min), 100% ethanol (2 min), xylene (2 × 2 min) and coverslipped using Mountex (Histolab)^41^. Brightfield images were captured using a Zeiss Axio Imager M1 Upright microscope. Control experiments were performed for all stainings by omitting either primary or secondary antibodies.

### Quantification of cell numbers and intensity measurements *in vivo*

The signal intensity of tyrosine hydroxylase fiber innervation of the midbrain aqueduct was quantified using Image J (http://rsb.info.nih.gov/ij/). Values are displayed as relative intensity of signal in controls. Quantification of the number of Ki67^+^ eGFP^+^ cells, using immunofluorescent staining coupled with confocal analysis, was performed at E12.5 (1/5 sections counted), E15.5 (1/6 sections counted) and E18.5 (1/7.5 sections counted) (Fig. 1Q–T). The total number of Ki67^+^ cells in the ventral midbrain regions were retrieved by using the counts and taken into account the total number of sections, the average diameter of the cells (10 μm), by performing the Abercrombie correction, according to P = A • (M/L + M) • n, where P is the average number of nuclear points, A the number of nuclei identified/section, M is the thickness of the section, L the average length of the cell (in μm) and n the number of sections. TH innervation of the midbrain aqueduct in Nurr1CKO and wild-type littermate mice was conducted on confocal projection images derived from z-stacks of identical depth using the Image J software (https://imagej.nih.gov/ij/).

For quantification of Ki67+ cells in the D2R KO mice analysis was conducted at E11.5 (1/5 sections counted) (Fig. 3E). To evaluate the number ventral eGFP^+^ pH3^+^ cells or eGFP^+^ BrdU^+^ cells, a Zeiss upright microscope was used. The number of cells was quantified under 63X magnification with the optical fractionator method on a systematic random sampling of every fifth sections along the rostro-caudal axis. To evaluate the number of BrdU^+^ cells in the prospective VTA and SN, 20x images encompassing the entire midbrain were generated and cell numbers within the TH^+^ area counted (number of BrdU^+^ cells/TH^+^ area measured in μm^3^) in 3 or 4 sections. To analyse the percentage of BrdU^+^TH^+^/BrdU^+^ cells, 40x confocal images along the entire Z-axis (12 μm) using 1 μm intervals were counted. The co-labeled cells were analyzed with Imarys software.

### *In situ* hybridization

*In situ* hybridization on sections was carried out essentially as previously described^42^ using a probe for Lmx1a^6^.

### Mouse primary midbrain cultures and immunocytochemistry

Brains from E11.5 mice were obtained and the midbrain region was dissected, mechanically dissociated, plated on poly-d-lysine (150,000 cells/cm^2^), and grown in serum-free N2 media consisting of F12/DMEM (1:1) with insulin (10 ng/ml), apo-transferrin (100 μg/ml), putrescine (100 μM), progesterone (20 nM), selenium (30 nM), glucose (6 mg/ml), BSA (1 mg/ml) and FGF2 (10 ng/ml). Cells were treated for 3 days *in vitro* with either dopamine (10 μM, Sigma), haloperidol (1 μM, Sigma), quinpirole (10μM, Tocris Bioscience), dihydrexidine (10 μM, Tocris Bioscience), muscimol (30 μM, Sigma) sulpiride (10 μM, Tocris Bioscience), SCH-23390 (10 μM, Santa Cruz Biotechnology), picrotoxin (30 μM, Tocris Bioscience) or media only and then processed for immunocytochemistry. For BrdU analysis, cells were treated with BrdU 1 hour after plating. After a further 2 days in culture, cells were treated for 30 minutes with 2N HCl, and processed for immunocytochemistry.

### Differentiation of mouse embryonic stem cells (mESCs)

R1 mESCs (Nagy’s lab, MSH, Toronto, Canada) were cultured and differentiated on PA6 stromal feeders (RCB1127, Riken BRC Cell Bank, Japan)^43^. Specifically, mESCs were plated at low density (100 cells/cm^2^) on a confluent layer of PA6 cells and were grown in Serum Replacement Medium with Noggin (300 ng/ml; R&D Systems) as previously described^44^. At day 5, 200 ng/ml Shh (R&D Systems) and 25 ng/mL Fgf8b (R&D Systems) were added to the medium. At day 8, the medium was switched to N2 medium containing Shh, Fgf8b, and Fgf2 (10 ng/ml, R&D Systems). At day 10 of differentiation the cells were pulsed with EdU (10 μM, Life Technologies). From day 11 of differentiation, Shh, Fgf8b and Fgf2 were removed from the N2 medium and replaced by BDNF (20 ng/ml, R&D Systems), GDNF (20 ng/ml, R&D Systems), and ascorbic acid (0.2 mM). Between day 8–15, cells were treated daily with either dopamine (10 μM), haloperidol (1 μM), quinpirole (10 μM), sulpiride (10 μM) or media only. At day 15, cells were fixed in 4% PFA and processed for immunocytochemistry as described below.

### Immunohistochemistry of mESC and primary cultures and *in vitro* quantifications

Cells were incubated for 1 h in blocking buffer prior to overnight incubation with the primary antibodies (Supplementary Table 1) at 4 °C. The coverslips were subsequently incubated in secondary antibodies (1:500, Life Technologies or Jackson Immunoresearch) for 1 h, and rinsed in PBS. EdU determinations were done according to Clik-it EdU kit (Life Technologies). DAPI or Hoechst (Sigma-Aldrich) was used for counterstaining. Each condition was analyzed in duplicates for three independent experiments (n = 3). Specifically, for the primary cultures, cells positive for each marker were counted in 8 consecutive fields/well. For the mESC-derived cultures, the number of TH^+^ and TH^+^ EdU^+^ cell in each well were quantified in 15 fields with an Olympus FV1000 confocal microscope.

### Statistical analysis

Prism (GraphPad software inc) and Statistica 8.0 (Statsoft) software were used for the statistical analyses. Experimental data was analysed using either student’s t test, 1-way ANOVA or 2-way ANOVA. All data are presented as mean ± SEM.

## Additional Information

**How to cite this article**: Hedlund, E. *et al.* Dopamine Receptor Antagonists Enhance Proliferation and Neurogenesis of Midbrain Lmx1a-expressing Progenitors. *Sci. Rep.* **6**, 26448; doi: 10.1038/srep26448 (2016).

## Supplementary Material

Supplementary Information
