# Supplementary material for: Dopamine Receptor Antagonists Enhance Proliferation and Neurogenesis of Midbrain Lmx1a-expressing Progenitors
Source: Sci Rep. 2016 Jun 1;6:26448. doi: 10.1038/srep26448 (PMC4887985; doi:10.1038/srep26448)
Supplement: Supplementary Information [file srep26448-s1.pdf]

Supplementary Information

Dopamine Receptor Antagonists Enhance Proliferation and Neurogenesis of Midbrain Lmx1a-expressing Progenitors

Eva Hedlund[1,2,*], Laure Belnoue[3,*], Spyridon Theofilopoulos[4], Carmen Salto[4], Chris Bye[5], Clare Parish[5], Qiaolin Deng[1,3], Banafsheh Kadkhodaei[1], Johan Ericson[3], Ernest Arenas[4,†], Thomas Perlmann[1,3,†], András Simon[3,†]

Supplementary Table 1. Antibodies used for immunofluorescence and immunohistochemistry.

Targets	Source	Catalogue #	Host species	Concentration	
				mouse tissues	*in vitro*
**BrdU**	Accurate	OBT0030G	rat	1:500	1:400
**D2R**	Millipore	ab5084	rabbit	1:250	-
**GFP**	Millipore	mab318	mouse	1:500	-
**GFP** (FITC)	Abcam	ab6662	goat	1:500	-
**GFP**	Abcam	ab6673	goat	1:500	-
**GFP**	Life Technologies	A6455	rabbit	1:1,000	-
**Ki67**	Novocastra	NCL-Ki67p	rabbit	1:1,000	-
**Lmx1a**	Millipore	ab10533	rabbit	1:500	-
**Nestin**	Pharmingen	556309	mouse	1:500	-
**Nestin**	Neuromics	GT15002	goat	1:250	-
**pHistone3**	Santa Cruz	sc-8656-R	rabbit	1:500	-
**Prominin**	MACS	W6B3C1	mouse	1/500	
**Sox1**	Millipore	AB15766	rabbit	1:2,000	-
**Sox2**	Millipore	AB5603	rabbit	1:1,000	-
**Sox2**	Santa Cruz	sc-17320	goat	1/250	
**Sox3**	Jonas Muhr lab	-	guineapig	1:1,000	-
**TH**	PelFreeze	P60101-150	sheep	1:1,000	-
**TH**	PelFreeze	P40101-150	rabbit	-	1:800-1:1,000
**TH**	Millipore	mab318	mouse	1:500	-
**TH**	Millipore	AB 152	rabbit	1:500	-

SUPPLEMENTARY FIGURE LEGENDS

Supplementary Figure 1. Ventral midbrain eGFP$^+$ ventricular cells maintain progenitor properties at E17.5 and E18.5. Immunofluorescent staining and confocal imaging showed that eGFP$^+$ cells were devoid of Sox1 at E18.5 (**A, B**), while expressing Sox2 (**C, D**) and Sox3 (**E, F**) at E17.5 and E18.5, respectively. Scale bar: 100 μM in E (applicable to A and C) and 20 μM in F (applicable to B and D).

Supplementary Figure 2. Ventral midbrain eGFP+ ventricular progenitors maintain progenitor properties in the adult animal. Immunofluorescent staining coupled with confocal imaging demonstrated that Sox2, which is present in the subventricular zone (SVZ) (**A**), was also present in eGFP$^+$ ventricular cells in the midbrain aqueduct (Aq) of 2 month-old animals (**B-D**). Sox3, which is present in the SVZ (**E**), was also present in a subset of eGFP$^+$ Aq ventricular cells (**G-I**). Cells in the SVZ express prominin (**J**) and a subset of eGFP$^+$ Aq ventricular cells also displayed prominin staining (**K-M**). Scale bar: 20 μM in J (applies to A and F) and 10 μM in M (applies to B-E, G-I and K, L)

Supplementary Figure 3. eGFP$^+$ ventricular cells express dopamine D2 receptors and are in close proximity to TH$^+$ fibers in the midbrain and hindbrain. Immunofluorescent staining and confocal imaging showed that ventricular cells lining the aqueduct expressed dopamine D2 receptor (D2R) at E18.5 (**A**) and this expression overlapped with eGFP, as shown by orthogonal view (**B**). Immunofluorescent analysis demonstrated that TH$^+$ fibers projected onto eGFP$^+$ cells in the midbrain ventricle, as shown in P0 mice (**C**, arrow heads indicate overlap between eGFP and TH; enlarged orthogonal view in **D**). TH$^+$ fibers from the locus coeruleus are in close proximity to eGFP$^+$ cells lining the fourth ventricle in the hindbrain, as shown in 3 month old mice (**E,F**). Scale bar: 25 μM in D (applicable to A and B), 200 μM in E and 25 μM in F (applicable to C).

Supplementary Figure 4. The effect of haloperidol treatment on the proliferation of eGFP$^+$ cells along the rostro-caudal axis in the midbrain: Rostro-caudal repartition of pH3$^+$eGFP$^+$ cells in control and haloperidol groups at E12.5-13.5, E13.5-14.5 and E14.5-16.5 **(A)**. Rostro-caudal repartition of BrdU$^+$eGFP$^+$ cells in control and haloperidol groups at E15.5-16.5, E16.5-17.5 **(B)**. Percentage of pH3$^+$eGFP$^-$ cells in cell layer lining the 3rd

ventricle in control and haloperidol groups for three developmental time points (**C**, E12.5-13.5, n=8 (Ctrl), n=8 (Hal); E13.5-14.5, n=11 (Ctrl), n=9 (Hal); E14.5-15.5, n=13 (Ctrl), n=10 (Hal), 2-way-ANOVA, treatment effect, P=0.000013). Haloperidol treatment at E15.5-E17.5 increases BrdU$^+$ cell density to the same extent in substantia nigra and ventral tegmental area (**D**, n=6 (Ctrl), n=6 (Hal), 2-way-ANOVA, treatment effect P=0,0008; region effect, P=0,53; treatment x region , P=0,92)

Supplementary Figure 5. TH$^+$ innervation in the midbrain at different time points along the rostro-caudal axis. Representative images of TH staining in the developing midbrain at E12.5-E13.5 (**A**), E13.5-E14.5 (**B**) and E15.5-E16.5 (**C**) showing the relatively higher density of TH$^+$ innervation surrounding the ventricle at more caudal levels. Scale bar: 50 μM.

Supplementary Figure 6. **Representative photomicrographs illustrating the effect of neurotransmitter receptor agonists and antagonists on cell proliferation and TH$^+$ cell numbers.** Representative pictures of BrdU$^+$ cells (**A**) and TH$^+$ cells (**B**) upon treatment with dopamine receptor and GABA receptor agonists and antagonists. Scale bar: 20 μM.

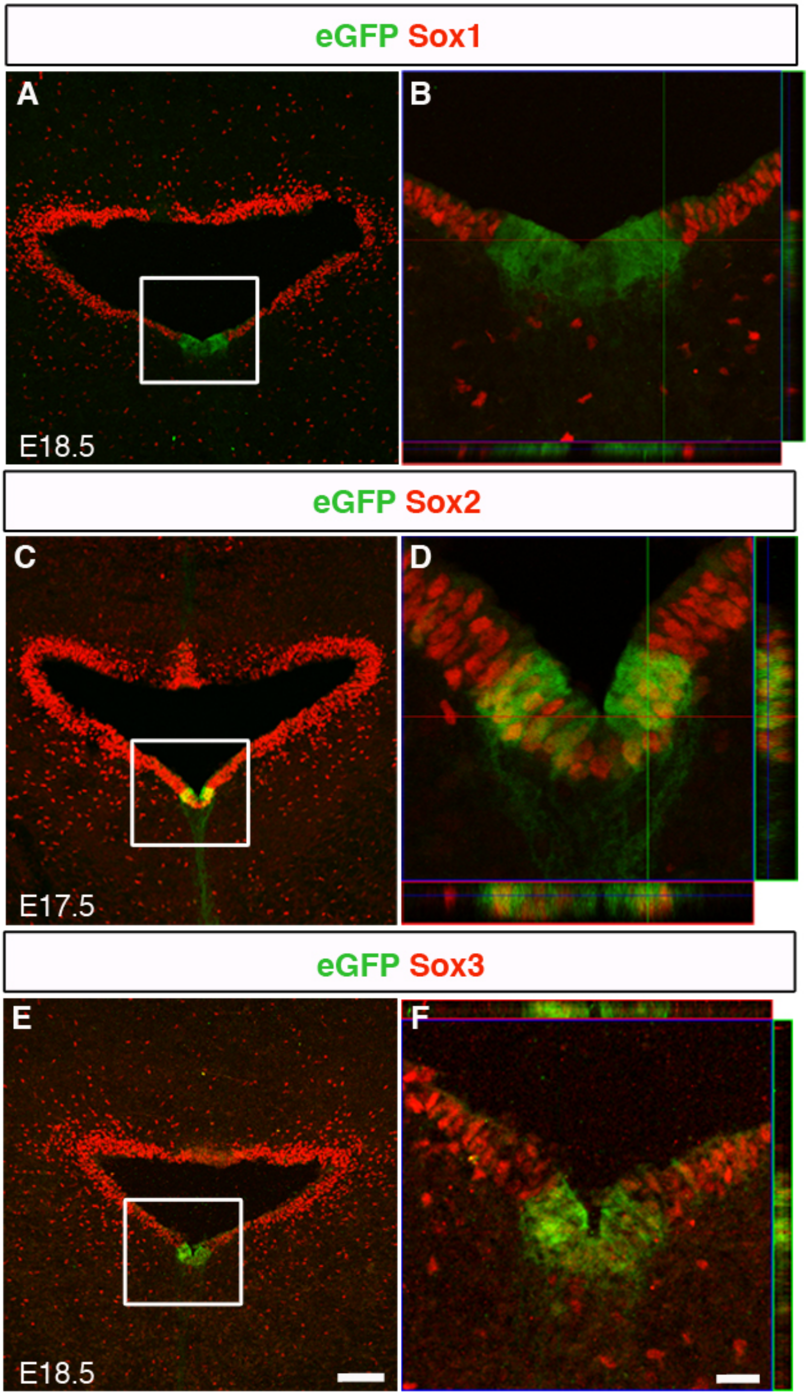

Supplementary Figure 1

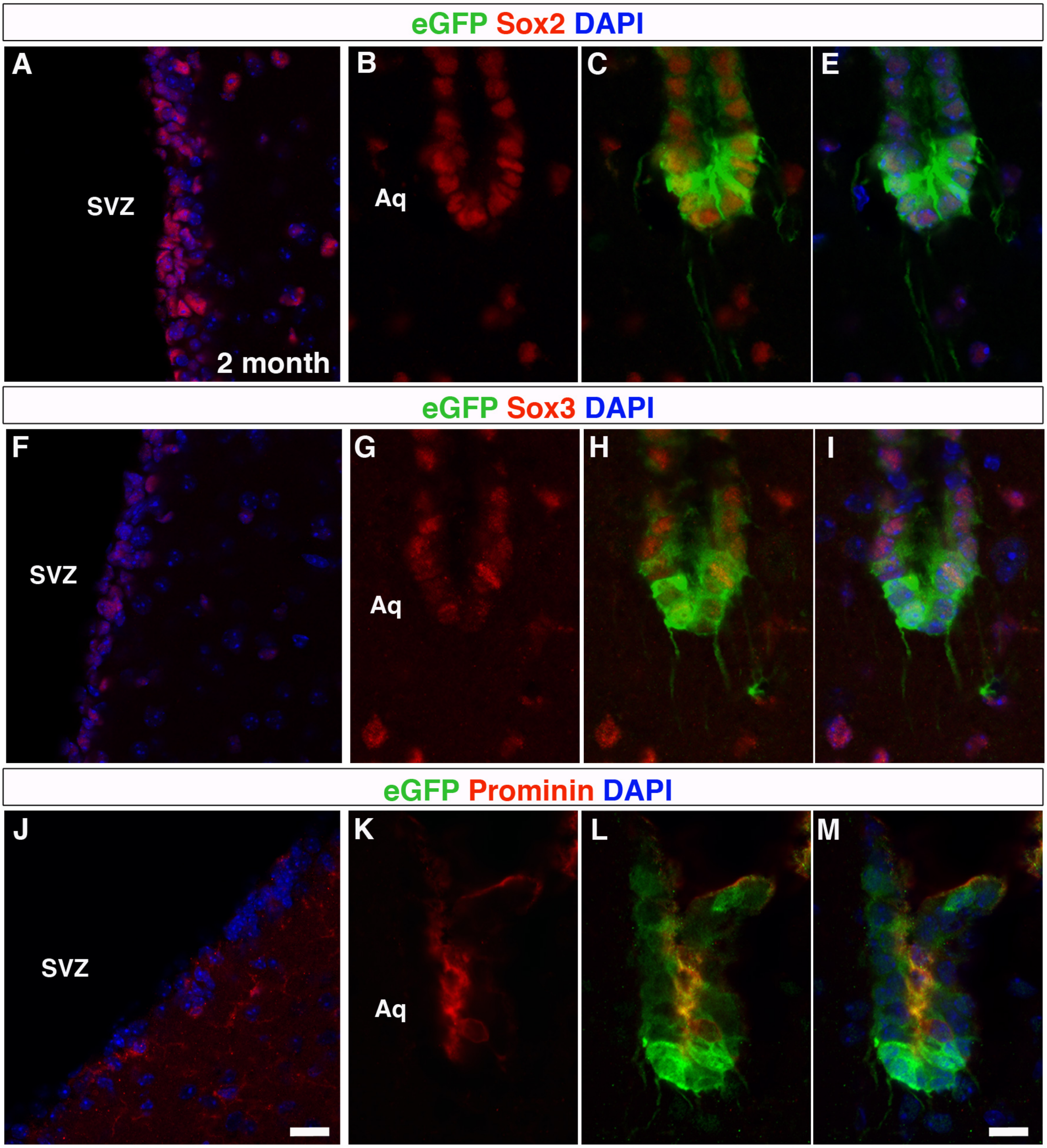

Supplementary Figure 2

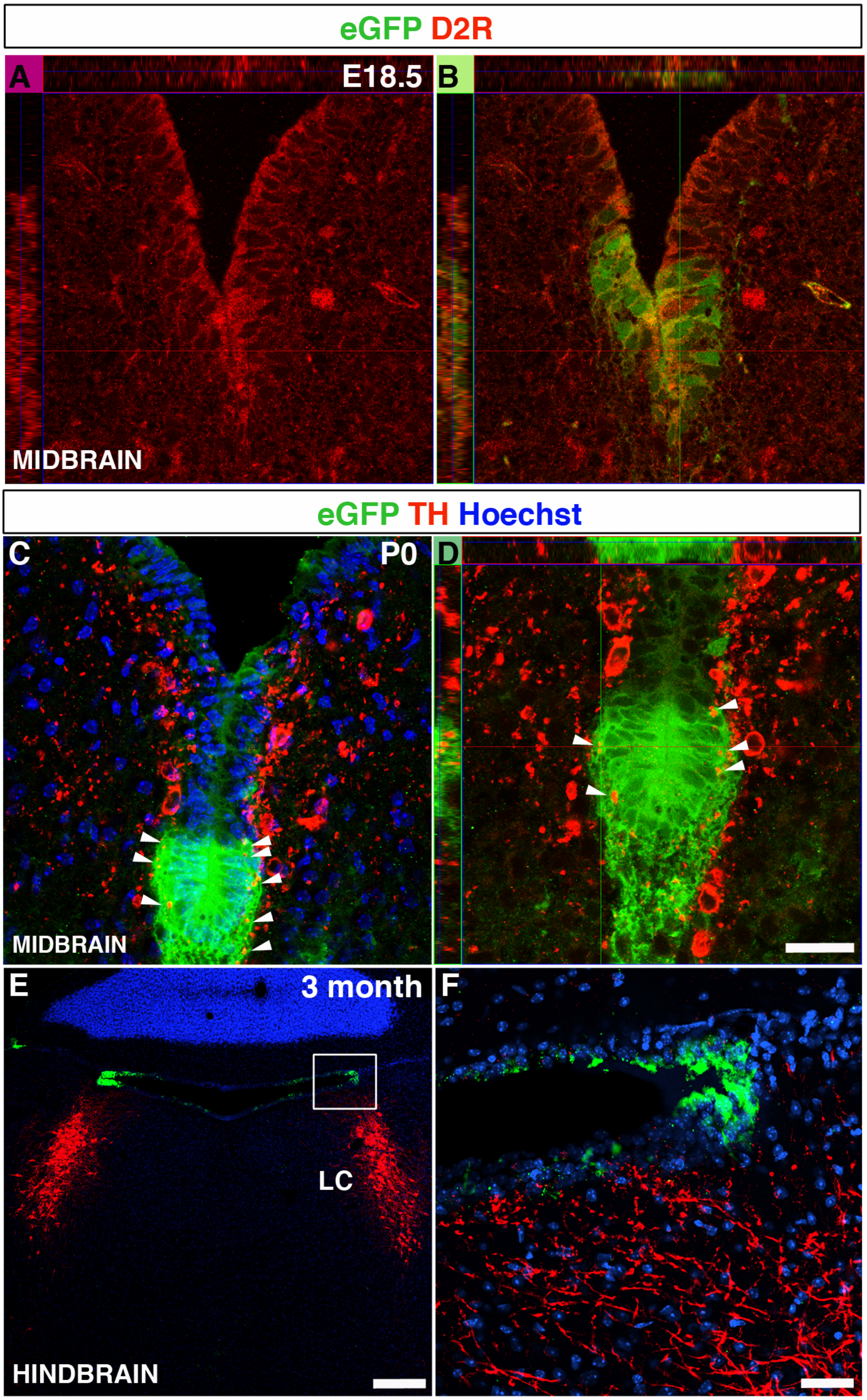

Supplementary Figure 3

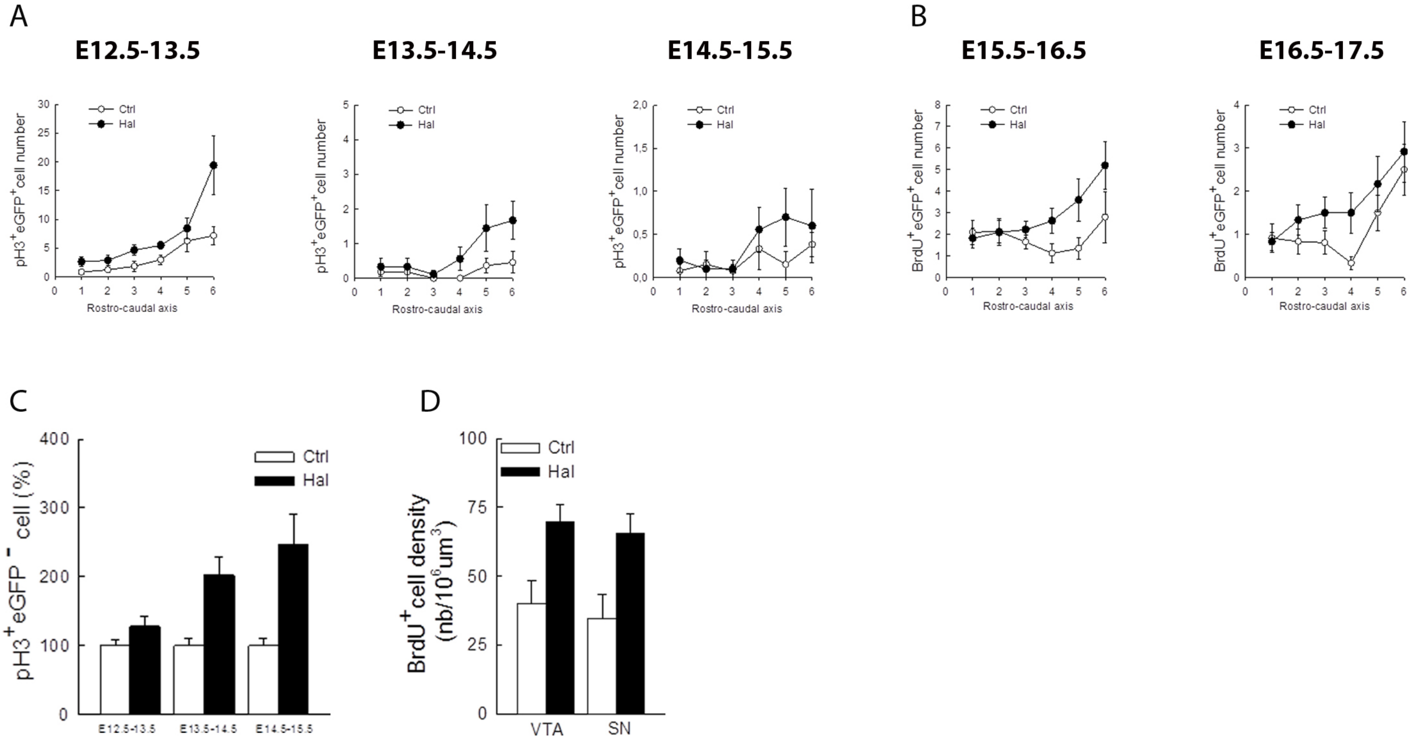

Supplementary Figure 4

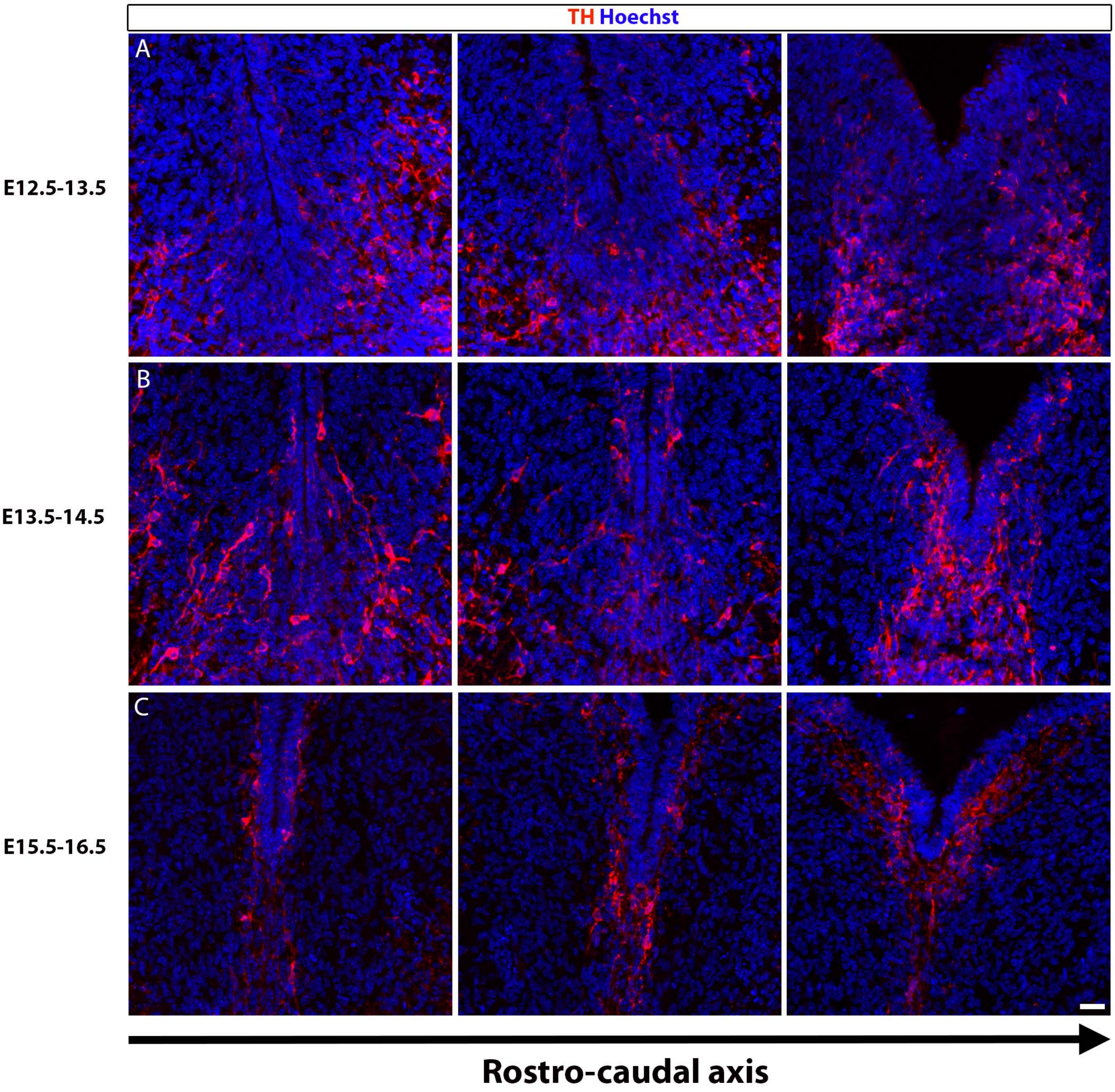

Supplementary Figure 5

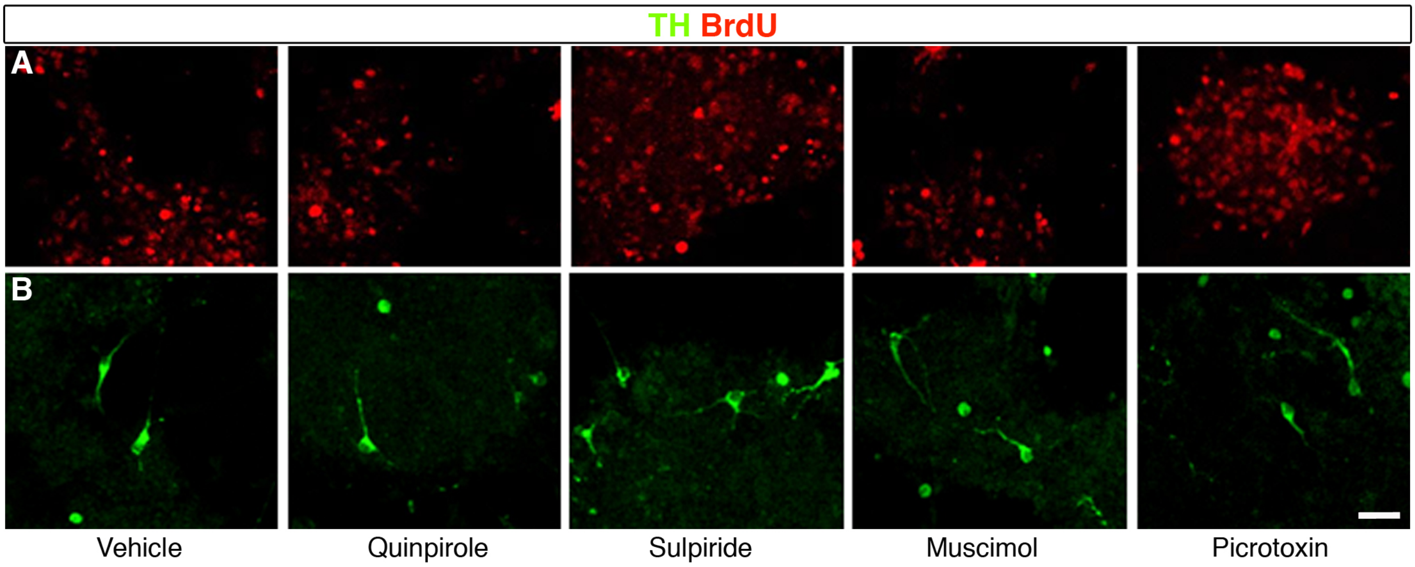

Supplementary Figure 6
